# Satellite Interference Source Direction of Arrival (DOA) Estimation Based on Frequency Domain Covariance Matrix Reconstruction

**DOI:** 10.3390/s23177575

**Published:** 2023-08-31

**Authors:** Jinjie Yao, Changchun Zhao, Jiansheng Bai, Yang Ren, Yangyang Wang, Jing Miao

**Affiliations:** 1 Shanxi Key Laboratory of Signal Capturing & Process, North University of China, Taiyuan 030051, China; yyyjinjie@163.com (J.Y.); sz202105023@st.nuc.edu.cn (C.Z.); s202205056@st.nuc.edu.cn (Y.R.); xinyunyu1992@163.com (Y.W.); 2 College of Mechanical Engineering, Suzhou University of Science and Technology, Suzhou 215009, China; jmiao@mail.usts.edu.cn

**Keywords:** DOA, satellite navigation interference source, coherent signal, spatial spectrum

## Abstract

Direction of arrival (DOA) estimation is an effective method for detecting various active interference signals during the satellite navigation process. It can be utilized for both interference detection and anti-interference applications. This paper proposes a DOA estimation algorithm for satellite interference sources based on frequency domain covariance matrix reconstruction (FDCMR) to address various types of active interference that may occur in the satellite navigation positioning process. This algorithm can estimate the DOA of coherent signals from multiple frequency points under low signal-to-noise ratio (SNR) conditions. The signals received from the array are transformed from the time domain to the frequency domain using a fast Fourier transform (FFT). The data corresponding to the frequency point of the target signal is extracted from the signal in the frequency domain. The frequency domain covariance matrix of the received array signals is reconstructed by utilizing its covariance matrix property. The spatial spectrum search method is used for the final DOA estimation. Simulation experiments have shown that the proposed algorithm performs well in the DOA estimation under low SNR conditions and also resolves coherency. Moreover, the algorithm’s effectiveness is verified through comparison with three other algorithms. Finally, the algorithm’s applicability is validated through simulations of various interference scenarios.

## 1. Introduction

Satellite navigation technology plays an essential role in various industries, as the world has entered a highly informationized era. Satellite navigation signals are affected by the ionosphere, atmospheric turbulence, and other factors during propagation, which can lead to severe attenuation [1,2,3,4], with minimum power levels as low as −160 dBW. Various types of interference [5] can affect the weak signals. Intentional interference signals received by the Global Navigation Satellite System (GNSS) can be classified into two types: jamming interference and spoofing interference [6,7]. Accurately locating the interference source and eliminating its impact is crucial in the field of satellite navigation and communication. Estimating the direction of satellite interference sources can help in identifying multipath interference signals and utilizing beamforming technology to eliminate interference [8,9,10], thus improving the signal’s robustness.

In recent years, research on interference monitoring systems in the field of satellite navigation has made some progress based on radio interference monitoring. The mainstream interference monitoring systems can be classified into airborne and ground monitoring platforms [11], with the aim of identifying the source of the interference [12]. Some researchers have designed ground interference monitoring platforms consisting of ground monitoring stations and monitoring vehicles, which can effectively measure the direction of interference signals and locate interference signals [13]. For airborne interference monitoring platforms, unmanned aerial vehicles are used to locate the interference source, which can eliminate the influence of terrain and effectively approach the interference source to a certain extent [14,15,16]. In the field of satellite navigation, research on interference source localization algorithms includes the utilization of adaptive filtering recursive least squares combined with generalized cross-correlation methods for delay estimation, as well as the use of TDOA algorithms for range localization of interference sources [17]. In the range localization method, the two-step localization approach is mostly used, and its localization accuracy is affected by parameter estimation. In addition, there is a direct location method (Despreading Direct Position Determination, DS-DPD [18]), which uses navigation signal characteristics to fuse TOA, Doppler frequency shift, and spreading sequences, aiming at scenarios with multiple spoofing interference sources. Although this method has high localization accuracy, it has significant computational complexity. With the development of array antenna technology [19], research on the direction finding and localization of satellite navigation interference sources has become a popular field. Currently, space filtering [20,21,22,23], adaptive beamforming [24,25,26], and DOA estimation technology [27,28,29] based on array antenna technology have begun to be applied in the field of satellite navigation interference monitoring and anti-interference.

Satellite navigation signals are affected by jamming interference signals, which disrupt the receiver’s normal functioning by transmitting high-power noise signals with a certain bandwidth. Additionally, these interference signals are easily detectable [30]. Spoofing interference signals simulate actual satellite signals and generate similar signals to mislead the receiver into tracking the fake signal. In addition, spoofing equipment can also forward actual signals, increase signal delay, and achieve the intended deception. Ultimately, these spoofing interference signals can cause errors in the receiver’s positioning results [31]. Regarding the characteristics of DOA estimation for satellite navigation interference sources, firstly, due to the similarity between spoofing signals and actual signals and the low SNR, DOA estimation algorithms need to work properly under low SNR conditions to measure the direction of the spoofing interference signal. Secondly, satellite navigation signals are multi-frequency signals, which can lead to multiple interference sources being distributed across multiple frequency bands. Furthermore, there may be multiple interference sources, including multipath interference, within a single frequency band. This requires DOA estimation algorithms to have the ability to work with multiple signals and frequencies as well as coherent signals. Considering the above-mentioned characteristics of DOA estimation for satellite navigation interference sources, existing classical DOA estimation algorithms such as the multiple signal classification (MUSIC) algorithm [32] and estimation of signal parameters via rotational invariance techniques (ESPRIT) algorithm [33] can only achieve high-precision direction finding under conditions of high SNR and multiple snapshots. The traditional MUSIC algorithm processes the array signal in the time domain and eigen-decomposes the signal covariance matrix to obtain the signal subspace corresponding to the signal component and the noise subspace orthogonal to the signal component. Utilizing the orthogonality of the two spaces, it estimates the direction of incidence of the signal. However, the algorithm is seriously affected by noise, resulting in poor accuracy of direction finding under low SNR conditions. In addition, since interference sources with multiple frequencies can affect the selection of inter-element spacing and thus affect the estimation accuracy of wave arrival direction, traditional algorithms rarely explore interference sources with multiple sources and frequencies. In recent years, some researchers have adopted sparse reconstruction methods based on compressive sensing theory for DOA estimation signal models with spatial sparsity characteristics, due to low SNR conditions [34]. This method has been applied to DOA estimation of interference sources in satellites and does not require prior knowledge of the number of interference sources. It achieves DOA estimation with fewer snapshots and improves estimation accuracy under low SNR conditions. In addition, this method can achieve high accuracy and resolution DOA estimation for multiple interference sources. However, this method does not consider the case where multiple interference sources are located at different frequency points. For multi-frequency signal DOA estimation, a sparse Bayesian learning-based multi-frequency DOA estimation algorithm has been proposed for passive radar [35], and a multi-frequency sparse reconstruction DOA algorithm based on mutual correlation array structure has also been proposed [36]. The above algorithms are based on optimization theory and changes in array structure to achieve multi-frequency signal DOA estimation. Regarding the DOA estimation of coherent signals, the spatial smoothing algorithm was initially proposed for uniform linear arrays [37] to achieve coherent signal separation by reducing the degrees of freedom of the array. In addition, a special array antenna model was used to reconstruct the Toeplitz matrix and achieve coherent signal separation [38]. Previous methods were unable to simultaneously perform DOA estimation on multi-frequency signals. They performed poorly in low SNR conditions and in estimating the DOA of coherent signals. They were unable to meet the requirements under all three aforementioned conditions simultaneously. The main contributions of this study are as follows: An algorithm for DOA estimation for satellite navigation interference sources under multi-frequency points and low signal-to-noise ratio conditions is proposed. The innovative time–frequency conversion processing of the array-received signal reduces the influence of noise and effectively improves the DOA estimation accuracy of the algorithm under the condition of low SNR, in addition to the excellent DOA estimation results for the same-frequency coherent signal. The feasibility of the algorithm is verified by simulation experiments.

The following sections will discuss our work in detail. Section 2 introduces the basic mathematical model of DOA estimation algorithms and the basic steps of our proposed FDCMR method. In Section 3, the feasibility of our FDCMR method is validated through simulation experiments, and a comparative analysis is conducted with three other algorithms to further demonstrate the superiority of our algorithm. Additionally, different interference scenarios are simulated for algorithm testing, such as scenarios with multiple coherent sources at the same frequency point, scenarios with multiple coherent sources at multiple frequency points, and scenarios with both coherent and incoherent sources at multiple frequency points. The final section provides a discussion and conclusion.

## 2. Algorithm

### 2.1. Introduction to the Proposed Algorithm

**Figure 1:**
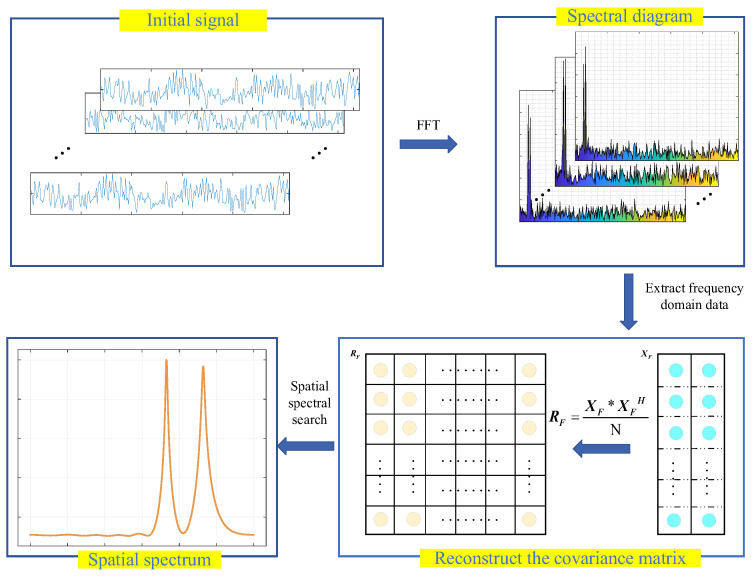
The block diagram of the FDCMR algorithm.

The paper presents an algorithm for DOA estimating based on frequency domain covariance matrix reconstruction (FDCMR). This approach is designed to address the detection of interfering sources’ DOA in satellite navigation scenarios, as shown in Figure 1. First, the array’s received signals undergo processing, and the signal is transformed from the time to frequency domain with fast Fourier transform (FFT). The frequency spectrum can identify the peak frequency of the target signal, allowing for the extraction of target signal peak data. This extraction process can efficiently eliminate noise influencing the data. Next, the extracted target signal data is processed to create the frequency domain covariance matrix of the received signals from the array. Finally, spatial spectrum search is utilized to obtain the DOA estimation value.

### 2.2. Signal Model

**Figure 2:**
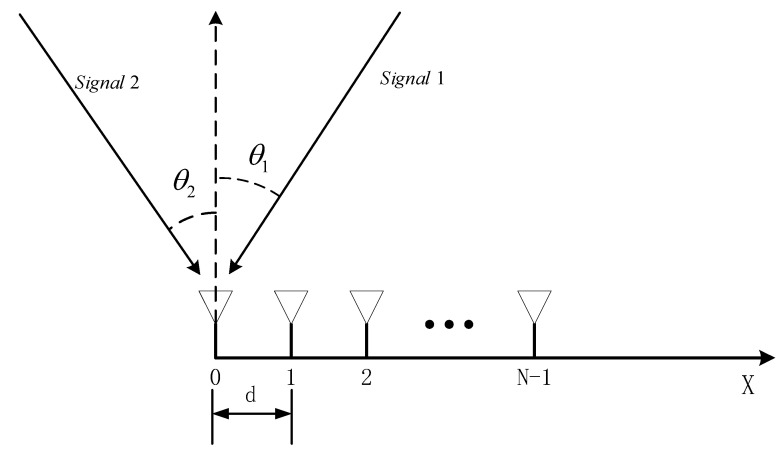
Schematic diagram of the array structure.

Assuming the receiving antenna array consists of N linearly arranged antennas, forming a uniform linear array with inter-antenna spacing of d=λ/2, as shown in Figure 2.

When Q signals $s_{q}(t)$ (with the wavelength corresponding to the maximum frequency signal being λ) impinge on the uniform linear array at angles $\theta_{q}$, q=1,…,Q with respect to the normal direction of the array, the signal received by the i-th antenna in the array can be represented as:

(1) $$x_{i}(t)=\sum_{q=1}^{Q}s_{q}(t)e^{j\frac{2\pi }{\lambda }(i-1)dsin\theta_{q}}+n_{i}(t)$$

where $n_{i}(t)$ is the Gaussian white noise signal.

The received signals by the array antennas can be represented in matrix form:

(2) x(t)=As(t)+n(t)

where x(t), s(t), n(t) are:

(3) $$x(t)={\left[x_{1}(t),x_{2}(t),\ldots ,x_{N}(t)\right]}^{T}$$

(4) $$n(t)={\left[n_{1}(t),n_{2}(t),\ldots ,n_{N}(t)\right]}^{T}$$

(5) $$s(t)={\left[s_{1}(t),s_{2}(t),\ldots ,s_{Q}(t)\right]}^{T}$$

where $A=[a(\theta_{1}),\ldots ,a(\theta_{Q})]$ is the array antenna steering vector, $a(\theta_{q})={\left[1,e^{j\frac{2\pi }{\lambda }dsin\theta_{q}},\ldots ,e^{j\frac{2\pi }{\lambda }(N-1)dsin\theta_{q}}\right]}^{T}$, M is the number of signal samples, also known as snapshots, and the received signal matrix for the array is an N × M dimensional matrix, represented as follows:

(6) $$\left[\begin{array}{c} x_{1}(t)\\ x_{2}(t)\\ \vdots \\ x_{N}(t) \end{array}\right]=\left[\begin{array}{cccc} 1 & 1 & \cdots & 1\\ e^{j\frac{2\pi }{\lambda }dsin\theta_{1}} & e^{j\frac{2\pi }{\lambda }dsin\theta_{2}} & \cdots & e^{j\frac{2\pi }{\lambda }dsin\theta_{Q}}\\ \vdots & \vdots & \ddots & \vdots \\ e^{j\frac{2\pi }{\lambda }(N-1)dsin\theta_{1}} & e^{j\frac{2\pi }{\lambda }(N-1)dsin\theta_{2}} & \cdots & e^{j\frac{2\pi }{\lambda }(N-1)dsin\theta_{Q}} \end{array}\right]\times \left[\begin{array}{c} s_{1}(t)\\ s_{2}(t)\\ \vdots \\ s_{Q}(t) \end{array}\right]+\left[\begin{array}{c} n_{1}(t)\\ n_{2}(t)\\ \vdots \\ n_{N}(t) \end{array}\right]$$

The covariance matrix of the received signal in the array is represented as

(7) $$R=E[x(t)x^{H}(t)]=AR_{s}A^{H}+R_{n}$$

where $R_{s}$, $R_{n}$ are the signal covariance matrix and noise covariance matrix, respectively.

The covariance matrix is obtained through maximum likelihood estimation in the actual data-processing process and is represented as follows:

(8) $$R=\frac{1}{M}\sum_{m=1}^{M}x(t_{m})x^{H}(t_{m})$$

### 2.3. Method in This Paper (FDCMR)

To achieve DOA estimation of interfering signals under multi-frequency and low SNR conditions, this paper introduces a method. This method utilizes FFT on the received signals, reconstructs the covariance matrix of the received signals, and performs a spatial spectrum search to achieve the final DOA estimation.

For the *N* × *M*-dimensional array-received signal x(t) in Equation (2), where each row corresponds to the signal of M snapshots from each antenna at the time, the received signal is subjected to FFT using the FFT formula, resulting in the frequency domain signal:

(9) $$X_{i}(k)=\sum_{m=1}^{M}x_{i}(m)e^{-j\frac{2\pi }{M}(m-1)(k-1)}$$

where M is the number of samples per signal snapshot, N represents the number of antennas, which is the number of rows in the received signal matrix x(t), k=1,2,…,M, i=1,2,…,N, the frequency domain array signal reception matrix is obtained through FFT:

(10) $$X_{FFT}={\left[X_{1},X_{2},\ldots ,X_{N}\right]}^{T}$$

(11) $$X_{i}=[X_{i}(1),X_{i}(2),\ldots ,X_{i}(M)]$$

(12) $$X_{FFT}=\left[\begin{array}{cccc} X_{1}(1) & X_{1}(2) & \cdots & X_{1}(M)\\ X_{2}(1) & X_{2}(2) & \cdots & X_{2}(M)\\ \vdots & \vdots & \ddots & \vdots \\ X_{N}(1) & X_{N}(2) & \cdots & X_{N}(M) \end{array}\right]$$

where $X_{FFT}$ represents the discrete spectral signal, which is composed of the noise spectrum and the signal spectrum. When there are Q different frequencies of incident signals, we can find Q peaks and corresponding useful signal spectra in the frequency domain received signals after FFT at each antenna. Other points are useless noise spectra.

In combination with Equation (2), since the matrix steering vectors are independent of time, Equation (16) can be written as

(13) $$X_{i}(k)=a_{n}\sum_{m=1}^{M}s_{i}(m)e^{-j\frac{2\pi }{M}(m-1)(k-1)}=a_{n}s_{i}(f)$$

where $a_{n}=[e^{j\frac{2\pi }{\lambda }(n-1)dsin\theta_{1}},e^{j\frac{2\pi }{\lambda }(n-1)dsin\theta_{2}},\ldots ,e^{j\frac{2\pi }{\lambda }(n-1)dsin\theta_{Q}}]$, i=1,…,Q, and n=1,…,N. $a_{n}$ is the n-th row of array antenna steering vector A, and $s_{i}(m)$ is the i column of target incident signal matrix s(t). So, the discrete spectral signals from the N antennas are completely identical, and the corresponding peak positions in the frequency domain for each incident signal are the same. By selecting the Q columns of the discrete spectral signals corresponding to Q spectral peaks from $X_{FFT}$ in Equation (10), we can form the frequency domain signal matrix $X_{F}$:

(14) $$X_{F}=[X_{F,1},\ldots ,X_{F,Q}]$$

(15) $$X_{F,q}={\left[X_{1}(f_{q}),X_{2}(f_{q}),\ldots ,X_{N}(f_{q})\right]}^{T}$$

where $f_{q}\in 1,2,\ldots ,M$, q=1,…,Q, so the formed $X_{F}$ is a N × Q-dimensional matrix. Next, we need to reconstruct R, which is the covariance matrix of the array-received signals. The R is a Hermitian matrix and $R=R^{H}$. The covariance matrix $R_{F}$ is reconstructed using the previously extracted frequency domain signal matrix $X_{F}$:

(16) $$R_{F}=\frac{X_{F}*X_{F}{}^{H}}{N}$$

where N represents the number of array antennas, after reconstructing the covariance matrix $R_{F}$, the target signal and noise are also mutually independent in the frequency domain. The frequency domain covariance matrix can be expressed as the sum of the autocorrelation of the frequency domain target signal and the frequency domain noise signal:

(17) $$R_{F}=R_{FS}+R_{FN}=(As(f)){\left(As(f)\right)}^{H}=AR_{s(f)}A^{H}+\sigma^{2}{}_{f}I$$

The signal s(t) is transformed into s(f) through FFT, where $\sigma^{2}{}_{f}$ represents the power component of the noise signal at the frequency points of the target signal in the frequency domain. $R_{s(f)}=s(f)s^{H}(f)$ is a diagonal matrix with diagonal elements representing the power components of the signal at the frequency points in the frequency domain.

Next, the eigenvalue decomposition can be performed on $R_{F}$:

(18) $$R_{F}=R_{FS}+R_{FN}=U_{F}\;\Sigma_{F}\;U_{F}{}^{H}$$

where $U_{F}=[e_{1},e_{2},\ldots ,e_{N}]$ is the matrix of eigenvectors, and the diagonal matrix $\Sigma_{F}$ is composed of the eigenvalues as follows:

(19) $$\Sigma_{F}=\left[\begin{array}{cccc} \lambda_{F1} & & & \\ & \lambda_{F2} & & \\ & & \ddots & \\ & & & \lambda_{FN} \end{array}\right]$$

The eigenvalues in the above equation satisfy the following relationship:

(20) $$\lambda_{F1}\geq \lambda_{F2}\geq \cdots \geq \lambda_{FQ}>\lambda_{FQ+1}=\cdots =\lambda_{FN}=\sigma^{2}{}_{f}$$

Define the following two diagonal matrices:

(21) $$\Sigma_{FS}=\left[\begin{array}{cccc} \lambda_{F1} & & & \\ & \lambda_{F2} & & \\ & & \ddots & \\ & & & \lambda_{FQ} \end{array}\right]$$

(22) $$\Sigma_{FN}=\left[\begin{array}{cccc} \lambda_{FQ+1} & & & \\ & \lambda_{FQ+2} & & \\ & & \ddots & \\ & & & \lambda_{FN} \end{array}\right]$$

where $\Sigma_{FS}$ is a diagonal matrix composed of the larger eigenvalues from the set of eigenvalues, and the corresponding eigenvectors $U_{FS}=[e_{1}\;e_{2}\;\cdots \;e_{Q}]$ form the frequency domain signal subspace. $\Sigma_{FN}$ is a diagonal matrix composed of the little eigenvalues from the set of eigenvalues, and the corresponding eigenvectors $U_{FN}=[e_{Q+1}\;e_{Q+2}\;\cdots \;e_{N}]$ form the frequency domain noise subspace. Therefore, the frequency domain covariance matrix $R_{F}$ can be decomposed as

(23) $$R_{F}=U_{FS}\Sigma_{FS}U_{FS}{}^{H}+U_{FN}\Sigma_{FN}U_{FN}{}^{H}$$

In the ideal situation, the frequency domain signal subspace and the frequency domain noise subspace are mutually orthogonal, meaning that the steering vectors are also orthogonal to the frequency domain noise subspace.

(24) $$a^{H}(\theta )U_{FN}=0$$

The presence of noise causes them to be not completely orthogonal; therefore, the implementation is achieved through a minimization optimization search, that is,

(25) $$\theta =\underset{\theta }{\arg\min}a^{H}(\theta )U_{FN}U^{H}{}_{FN}a(\theta )$$

Therefore, the spectral estimation formula is given by

(26) $$P_{FDCMR}=\frac{1}{a^{H}(\theta )U_{FN}U^{H}{}_{FN}a(\theta )}$$

The above content provides a detailed introduction to the DOA estimation algorithm based on the reconstruction of the frequency domain covariance matrix proposed in this paper. The following Table 1 summarizes the main steps of the FDCMR:

## 3. Results

To validate the performance of the DOA estimation algorithm, the root mean square error (RMSE) is used to evaluate the algorithm performance:

(27) $$RMSE=\frac{1}{Q}\sum_{q=1}^{Q}\sqrt{\frac{1}{D}\sum_{i=1}^{D}{\left({\hat{\theta }}_{q,i}-\theta_{q}\right)}^{2}}$$

where ${\hat{\theta }}_{q,i}$ represents the angle of the q-th incident signal in the i-th Monte Carlo experiment, and a total of D = 300 Monte Carlo experiments are conducted. Q represents the number of incident signal sources. We assume that there are Q = 2 incident signals, with signal frequencies of $f_{1}$ = 1575.42 MHz for GPS navigation satellite L1 frequency and $f_{2}$ = 1268.52 MHz for BeiDou navigation satellite B3 frequency. The incident signal angles are $\theta_{1}=20^{\circ }$ and $\theta_{2}=50^{\circ }$. The number of array antennas is N=8, and the number of snapshots is M=256.

### 3.1. Experiment 1

**Figure 3:**
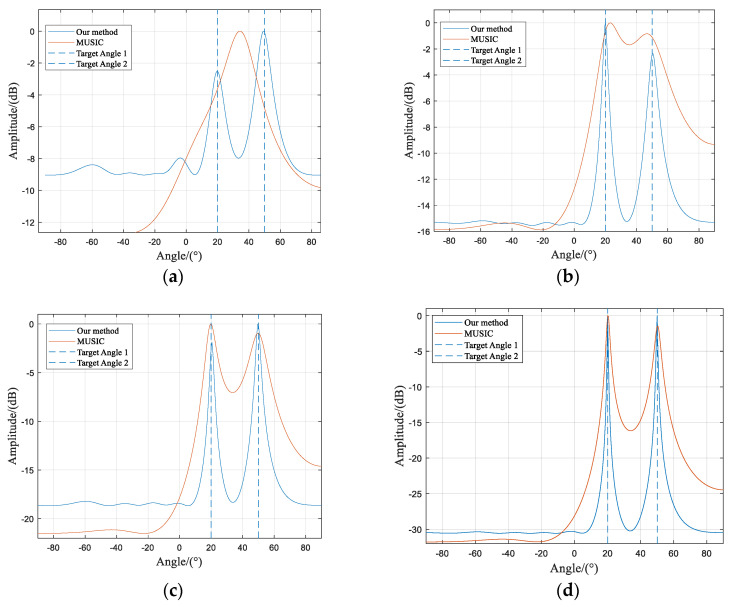
Comparison of two algorithms on spatial spectra under different SNR conditions. (**a**) SNR = −15 dB; (**b**) SNR = −5 dB; (**c**) SNR = 0 dB; (**d**) SNR = 10 dB.

A comparison is presented between the spatial spectra of the algorithm proposed in this paper and those of the traditional MUSIC algorithm under low SNR conditions. The conditions of SNR at −15 dB, −5 dB, 0 dB, and 10 dB are compared individually, as demonstrated by Figure 3.

From Figure 3a, it can be observed that at an SNR of −15 dB, the traditional MUSIC algorithm fails to resolve the two incident signals correctly, and the DOA estimation results are erroneous. However, the algorithm proposed in this paper can clearly distinguish between the two incident signals. Both algorithms presented in Figure 3b–d successfully resolve the two signals. However, the traditional MUSIC algorithm exhibits wider spatial spectrum peaks, which indicates lower signal resolution compared to the method proposed in this paper.

### 3.2. Experiment 2

**Figure 4:**
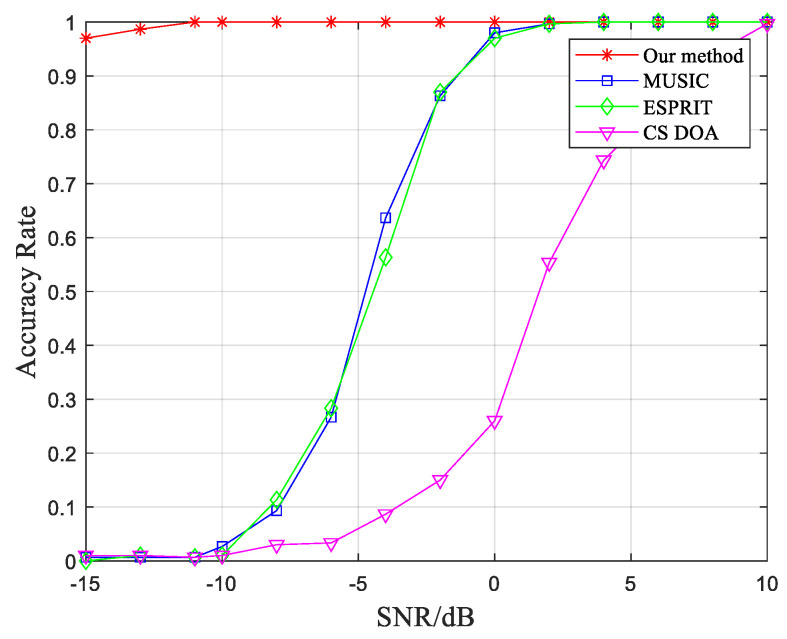
Comparison of DOA estimation accuracy of different methods under different SNR conditions.

To validate the success probability of the algorithm for DOA estimation under various SNR conditions, a series of Monte Carlo experiments are conducted. The SNR is increased gradually from −15 dB to 10 dB with an interval of 2 dB, and each experiment is repeated 300 times. For reference, the success probability of three other methods, namely the conventional MUSIC, ESPRIT, and compressed sensing-based orthogonal matching pursuit algorithm [39], are also calculated under different SNR conditions. The experimental results are shown in Figure 4.

From Figure 4, it can be observed that the proposed method achieves higher accuracy in DOA estimation under low SNR conditions. In contrast, the traditional MUSIC and ESPRIT algorithms experience a rapid decline in DOA estimation accuracy when the SNR falls below 0 dB. When the SNR drops below −10 dB, these two algorithms become practically ineffective. On the other hand, the compressed sensing-based DOA estimation algorithm offers the advantage of reduced computational time since it only selects a single snapshot signal for DOA estimation. However, its accuracy is compromised under low SNR conditions, resulting in a significant decrease in success probability compared to the other three estimation algorithms.

### 3.3. Experiment 3

**Figure 5:**
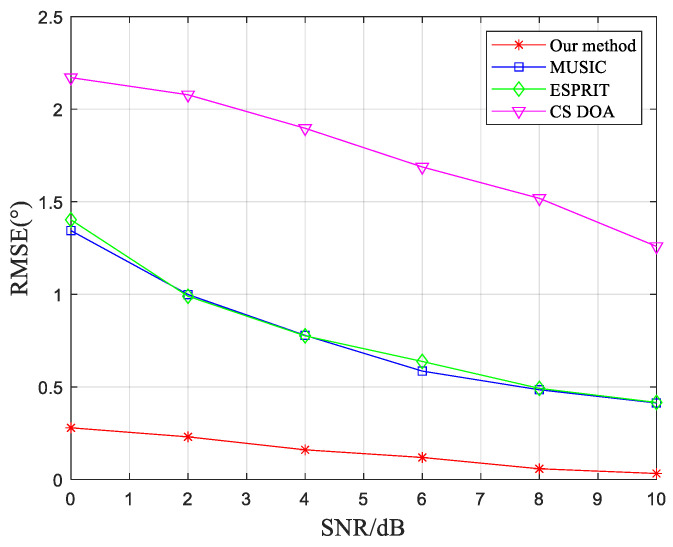
Comparison of RMSE for DOA estimation of different algorithms under different SNR conditions.

In addition to the success rate of DOA estimation, the accuracy of the algorithm’s DOA estimation is also a crucial factor. The RMSE mentioned in Equation (27) is used as the performance evaluation criterion for the algorithm. The experimental conditions are the same as those used to validate the success probability of DOA estimation in the previous analysis. Since the algorithms in the control group exhibit lower accuracy in DOA estimation under low SNR conditions, the RMSE is calculated for SNR values ranging from 0 dB to 10 dB. Experimental results are presented in Figure 5.

From Figure 5, it is evident that the proposed method exhibits a smaller RMSE compared to the traditional MUSIC algorithm, ESPRIT algorithm, and compressed sensing-based DOA estimation algorithm. This significant improvement in DOA estimation performance can meet the requirements of practical applications.

**Figure 6:**
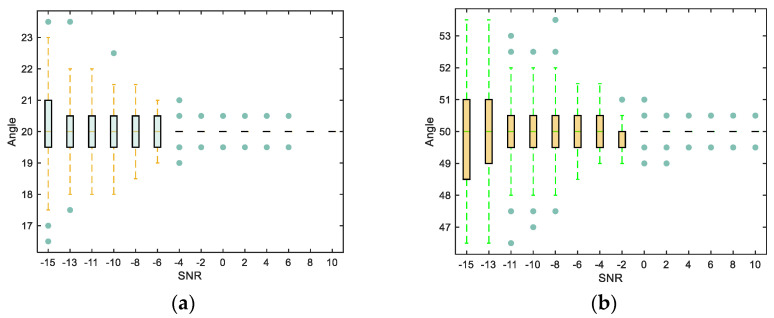
Statistical chart of DOA estimation results using our algorithm under different SNR conditions. Boxes represent the 25th and 75th percentiles, and the central line represents the median. (**a**) Incident angle $\theta_{1}=20^{\circ }$, (**b**) Incident angle $\theta_{2}=50^{\circ }$.

To verify the higher accuracy of the proposed algorithm in DOA estimation under low SNR conditions, the SNR is set in the range of −15 dB to 10 dB. In each simulation experiment, the Monte Carlo experiment is repeated 300 times, and a box plot is generated to visualize the statistical distribution of the DOA estimation results. From Figure 6, it can be observed that at an SNR of −15 dB, the DOA estimation results are within the theoretical range of ±3°, with the majority concentrated within an error range of ±1°. As the SNR increases, the error range gradually narrows.

### 3.4. Experiment 4

**Figure 7:**
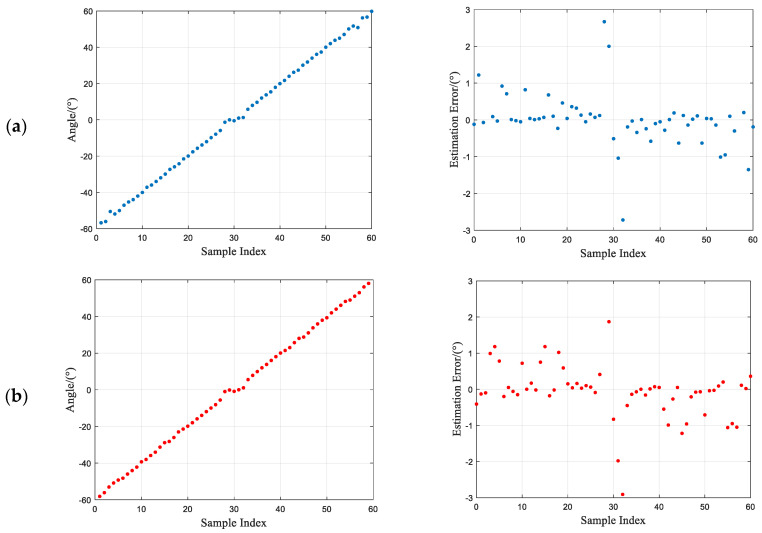
DOA estimation results and errors for different incident signal angles. (**a**) DOA estimation results and errors for incident signal 1. (**b**) DOA estimation results and errors for incident signal 2.

To further validate the robustness of the algorithm in DOA estimation for different angles, the experiment analyzes the change in the incident direction of two signals with different frequencies, ranging from −60° to 60° with an interval of 2°. The SNR is set to −10 dB. The DOA estimation results are shown in Figure 7.

From Figure 7, it can be observed that the proposed algorithm exhibits strong robustness. Within the range of [−60°, 60°], even under low SNR conditions, the error remains within a range of ±2°, with a few points reaching ±3°. In this experiment, a relatively low SNR is set, which introduces significant noise. If the interference from noise is further reduced, the error in the experimental results may decrease even further.

To validate that the algorithm is suitable for DOA estimation applications in satellite interference scenarios, considering the characteristics of low SNR, multiple frequency points, and phase coherence, simulations are conducted under the following application scenarios.

### 3.5. Scenario 1: Multiple Coherent Sources at the Same Frequency

The scenario assumes there are Q=2 interference signals, both of which are coherent signals with a frequency of $f_{1}$ = 1268.52 MHz, corresponding to the BeiDou Navigation Satellite System (BDS) signal at the B3 frequency point. The incident angles of the two signals are $\theta_{1}=20^{\circ }$ and $\theta_{2}=50^{\circ }$, respectively. The array consists of N=8 antenna elements, and the number of snapshots is M=256. The SNR is −10 dB.

**Figure 8:**
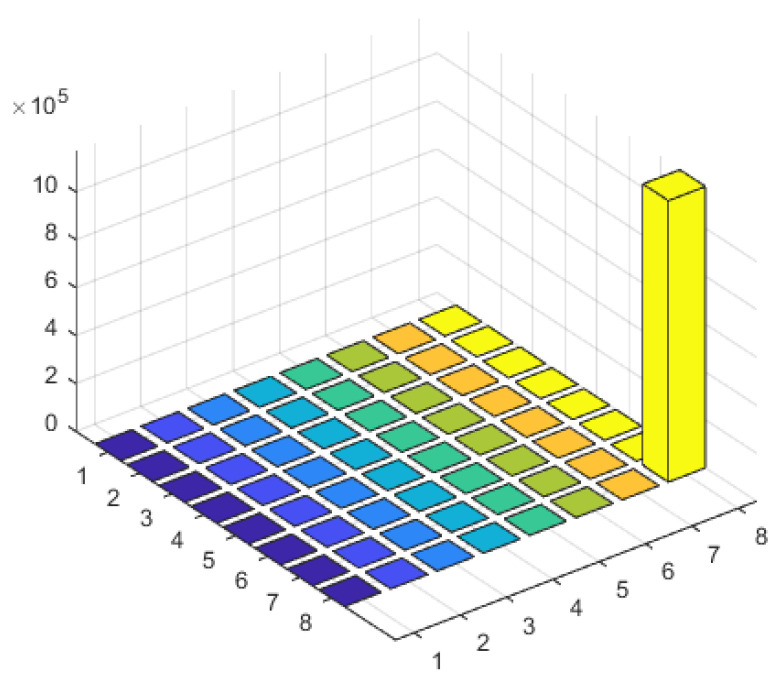
Characteristic value matrix diagram of interference scenario 1.

**Figure 9:**
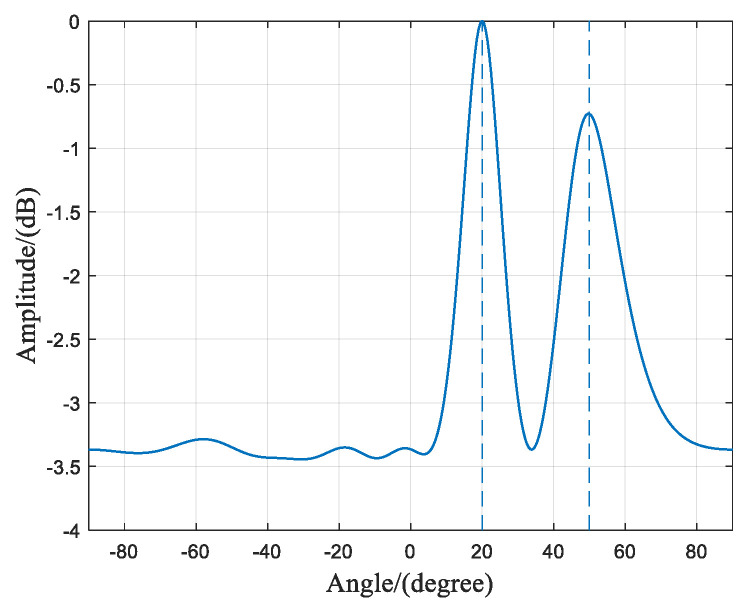
The spatial spectrum of interference scenario 1.

From Figure 8, it can be observed that due to the interference sources being coherent signals, the received signal’s frequency domain covariance matrix is no longer full rank. In this case, rank{R}=Q−1, indicates a rank-deficient matrix. When performing the eigen value decomposition of the covariance matrix, only Q−1 signal eigenvectors can be obtained. In this experiment, Q=2 is set, so only one eigenvalue of the covariance matrix is non-zero. Consequently, when constructing the signal subspace using the eigenvectors, the complete signal subspace cannot be obtained since its dimension is smaller than the number of sources. However, by performing time–frequency transformations on the received signals from the array, the data at the target frequency point already contains information about the two coherent sources with the same frequency but different incident angles. Therefore, even if the dimension of the signal subspace is smaller than the number of sources, the proposed algorithm can still distinguish between the two incident directions, as shown in Figure 9’s spatial spectrum.

### 3.6. Scenario 2: Multiple Coherent Sources at Multiple Frequency Points

Assuming there are Q=4 interference signals, the first two interference signals are coherent signals with a frequency of $f_{1}$ = 1575.42 MHz, corresponding to the GPS navigation satellite signal at the L1 frequency point. The incident angles of these two signals are $\theta_{1}=20^{\circ }$ and $\theta_{2}=50^{\circ }$, respectively. The other two interference signals are also coherent signals with a frequency of $f_{2}$ = 1268.52 MHz, corresponding to the BDS signal at the B3 frequency point. The incident angles of these two signals are $\theta_{3}=-20^{\circ }$ and $\theta_{4}=-50^{\circ }$, respectively. The array consists of N=8 antenna elements, and the number of snapshots is M=256. The SNR is −10 dB.

**Figure 10:**
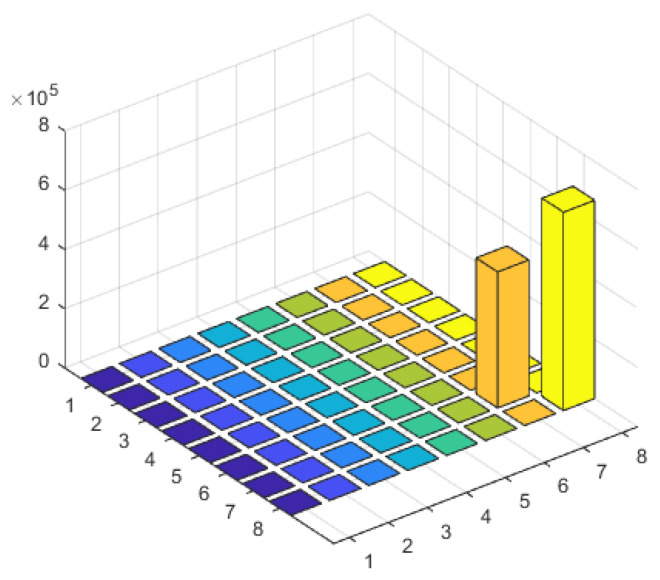
Characteristic value matrix diagram of interference scenario 2.

**Figure 11:**
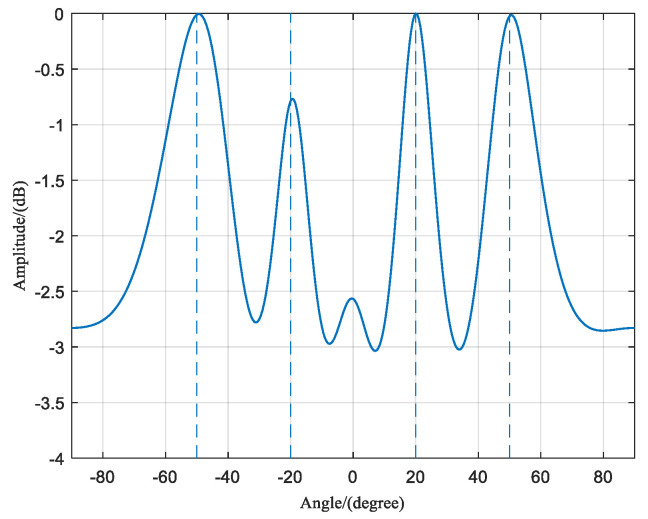
Spatial spectrum of interference scenario 2.

When there are interference signals at two frequency points, each with two coherent signals, the rank of the received signal covariance matrix can be determined as 2, according to the formula. Therefore, in Figure 10, the eigenvalue matrix of the frequency domain covariance matrix shows two eigenvalues, corresponding to the number of frequency points. Figure 11 shows the spatial spectrum of the DOA estimation using the proposed algorithm. It clearly distinguishes the two coherent signals in the two frequency points and achieves accurate DOA estimation with minimal error.

### 3.7. Scenario 3: Coexistence of Incoherent and Coherent Sources at Multiple Frequency Points

Assuming there are Q=3 interference signals, the first two interference signals are coherent signals with a frequency of $f_{1}$ = 1575.42 MHz, corresponding to the GPS navigation satellite signal at the L1 frequency point. The incident angles of these two signals are $\theta_{1}=20^{\circ }$ and $\theta_{2}=50^{\circ }$, respectively. The last interference signal is an incoherent signal with a frequency of $f_{2}$ = 1268.52 MHz, corresponding to the BDS signal at the B3 frequency point. The incident angle of this signal is $\theta_{3}=-20^{\circ }$. The array consists of N=8 antenna elements, and the number of snapshots is M=256. The SNR is −10 dB.

**Figure 12:**
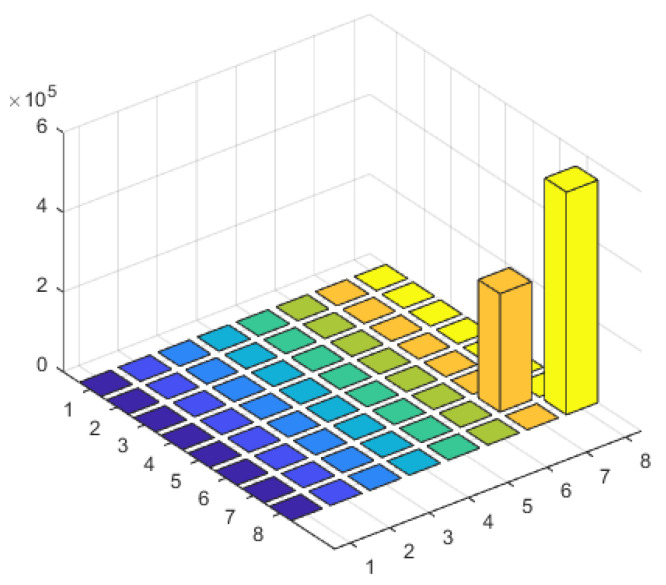
Characteristic value matrix diagram of interference scenario 3.

**Figure 13:**
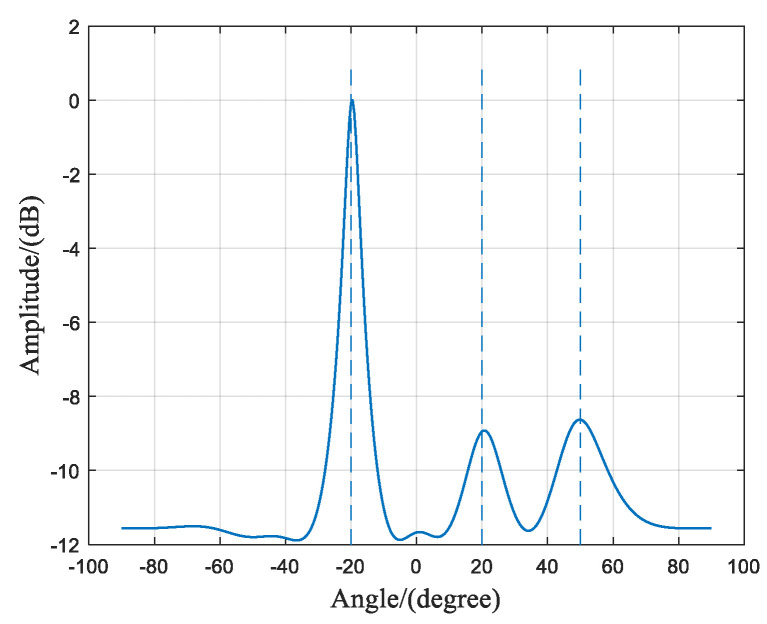
The spatial spectrum of interference scenario 3.

In the case where there are interference signals at two frequency points, with one frequency point containing two coherent signals, the frequency domain covariance matrix of the received signal is rank{R}=Q−1=2. Figure 12 shows two peaks, which further confirms that the eigenvalues of the frequency domain covariance matrix of the array-received signals are related to the number of target frequency points. Figure 13 represents the spatial spectrum of the DOA estimation using the proposed algorithm. Despite the interference signals at 20° and 50° being two coherent signals at the same frequency point, their amplitudes in the spatial spectrum are relatively smaller compared to the signals at the other frequency point. However, this does not affect the accuracy of the DOA estimation results.

The feasibility of this method in the estimation of DOA for multi-frequency coherent signals has been verified through simulations involving scenarios with multiple coherent sources at the same frequency, multiple coherent sources at multiple frequencies, and the coexistence of multiple coherent sources and incoherent sources at multiple frequencies. By using the same frequency as the satellite navigation signals, the method’s feasibility in the estimation of DOA for multifrequency coherent signals has been confirmed. Moreover, in the scenario of DOA estimation for satellite navigation interference sources, the presence of multi-frequency coherent signals, such as multipath interference signals, suppression interference signals at different frequencies, and deception interference signals, should also be considered. Therefore, this method is promising for the estimation of DOA in satellite navigation interference source scenarios.

## 4. Discussion

In this paper, the FDCMR method is proposed to address the following challenges in the field of satellite interference source DOA estimation. Spoofing interference signals are similar to the original navigation satellite signals and exhibit high levels of camouflage. Thus, the DOA estimation algorithm must be able to perform robustly even in low SNR conditions in order to effectively handle such interference signals. Multiple frequency point interference signals and coherent signals exist in satellite navigation systems. The DOA estimation algorithm should be capable of accurately estimating the DOA of multiple coherent signals at various frequency points.

This paper presents an algorithm for fast and accurate estimation of DOA for multi-frequency coherent signals under low SNR conditions. The algorithm utilizes FFT on the received signal matrix to extract the target signal at the corresponding frequencies, known as frequency domain peak signals. It reconstructs the data covariance matrix and uses spatial spectrum search to plot the spatial spectrum, thereby achieving DOA estimation of the target signal. The algorithm applies frequency domain filtering by extracting the target frequency data, discarding irrelevant frequency data, reducing noise interference, and improving the algorithm’s performance under low SNR conditions. FDCMR can be used for DOA estimation in satellite navigation positioning, even in the presence of various active interferences such as jamming and spoofing. The feasibility and effectiveness of this algorithm are verified through simulation experiments. Compared to other DOA estimation algorithms, this method has significant advantages in low SNR conditions and is crucial for addressing various active interferences in satellite navigation.

Currently, the focus of this work is mainly on DOA estimation based on one-dimensional linear arrays. However, to achieve an estimation of azimuth and elevation angles of multi-frequency signals within a spatial volume, it is necessary to construct a two-dimensional planar array structure. In future work, efforts will be made to extend the method proposed in this paper to two-dimensional DOA estimation algorithms.

## Figures and Tables

**Table 1:** Main steps of the FDCMR.

Step	Description
Step 1	Perform FFT on each row of the received signal matrix x(t) to obtain frequency domain signals $X_{FFT}\in {\mathbb{C}}^{N\times M}$. Each row of the matrix x(t) corresponds to the received signal data from each antenna using Equation (9).
Step 2	Extract Q peaks from each row of the frequency spectrum of $X_{FFT}$ to form $X_{F}\in {\mathbb{C}}^{N\times Q}$.
Step 3	Reconstruct the covariance matrix $R_{F}$ of the received signals using Equation (16).
Step 4	Perform eigenvalue decomposition on the covariance matrix $R_{F}$ to calculate the frequency domain noise subspace $U_{FN}$.
Step 5	Perform peak search using Equation (26) to obtain the estimated DOA result.
Input:	Received signal matrix $x(t)\in {\mathbb{C}}^{N\times M}$, Q interfering sources, N receiving antennas, M sampling snapshots

## Data Availability

All data and code will be made available on request to the author’s email with appropriate justification.
